# Supplementary material for: Crawling toward obsolescence: The extended lifespan of amylase for pancreatitis
Source: PLoS One. 2023 Dec 21;18(12):e0296180. doi: 10.1371/journal.pone.0296180 (PMC10734915; doi:10.1371/journal.pone.0296180)
Supplement: Attachment — Submitted filename: Reviewer comments and responses Amylase 2023 - RGH.docx [file pone.0296180.s002.docx]

Dear Editor,

Thank you for the opportunity to make revisions to the original manuscript. Please find the summary of the revisions here. Our responses to reviewer comments are in bold.

Cheers,

Naga Kanaparthy

Andrew Loza

Ronald Hauser

# Journal Requirements:

*1. When submitting your revision, we need you to address these additional requirements.*

*Please ensure that your manuscript meets PLOS ONE's style requirements, including those for file naming. The PLOS ONE style templates can be found at* 
[*https://journals.plos.org/plosone/s/file?id=wjVg/PLOSOne_formatting_sample_main_body.pdf*](https://journals.plos.org/plosone/s/file?id=wjVg/PLOSOne_formatting_sample_main_body.pdf) *and* 
[*https://journals.plos.org/plosone/s/file?id=ba62/PLOSOne_formatting_sample_title_authors_affiliations.pdf*](https://journals.plos.org/plosone/s/file?id=ba62/PLOSOne_formatting_sample_title_authors_affiliations.pdf)

**- We have updated the style requirements as per the template in the title page.**

**- We have updated the style requirements in the body of the manuscript. Font sizes of headings have been changed 18.**

**- The figure image has been removed from manuscript and will be uploaded separately. We kept the title and legend at the end of the Results section.**

2. *In your Data Availability statement, you have not specified where the minimal data set underlying the results described in your manuscript can be found. PLOS defines a study's minimal data set as the underlying data used to reach the conclusions drawn in the manuscript and any additional data required to replicate the reported study findings in their entirety. All PLOS journals require that the minimal data set be made fully available*

**- We will submit an Excel document as a supplemental document. This file contains the raw aggregate data used for this study.**

*3. Please review your reference list to ensure that it is complete and correct. If you have cited papers that have been retracted, please include the rationale for doing so in the manuscript text, or remove these references and replace them with relevant current references. Any changes to the reference list should be mentioned in the rebuttal letter that accompanies your revised manuscript. If you need to cite a retracted article, indicate the article’s retracted status in the References list and also include a citation and full reference for the retraction notice.*

**We have reviewed the reference list for accuracy. There are no retracted articles that have been cited here. The references have been listed in chronological order of appearance in the body of the article. The Endnote library has been been cross-checked. The references are in Vancouver style.**

Reviewers' comments:

**Reviewer's Responses to Questions**

**Comments to the Author**

*1. Is the manuscript technically sound, and do the data support the conclusions?

The manuscript must describe a technically sound piece of scientific research with data that supports the conclusions. Experiments must have been conducted rigorously, with appropriate controls, replication, and sample sizes. The conclusions must be drawn appropriately based on the data presented.*

*Reviewer #1: Partly*

*Reviewer #2: Partly*

2. Has the statistical analysis been performed appropriately and rigorously?

Reviewer #1: Yes

Reviewer #2: N/A

3. Have the authors made all data underlying the findings in their manuscript fully available?

The [PLOS Data policy](https://nam12.safelinks.protection.outlook.com/?url=http%3A%2F%2Fwww.plosone.org%2Fstatic%2Fpolicies.action%23sharing&data=05%7C01%7Cnaga.kanaparthy%40yale.edu%7C520a6216d43f4ee85ba908dbe9e226d4%7Cdd8cbebb21394df8b4114e3e87abeb5c%7C0%7C0%7C638360929745966510%7CUnknown%7CTWFpbGZsb3d8eyJWIjoiMC4wLjAwMDAiLCJQIjoiV2luMzIiLCJBTiI6Ik1haWwiLCJXVCI6Mn0%3D%7C3000%7C%7C%7C&sdata=X0H02eVw9eaIFVwu4xv%2FWxiShWzve3H%2FExe8VR%2FjEY8%3D&reserved=0) requires authors to make all data underlying the findings described in their manuscript fully available without restriction, with rare exception (please refer to the Data Availability Statement in the manuscript PDF file). The data should be provided as part of the manuscript or its supporting information, or deposited to a public repository. For example, in addition to summary statistics, the data points behind means, medians and variance measures should be available. If there are restrictions on publicly sharing data—e.g. participant privacy or use of data from a third party—those must be specified.

Reviewer #1: Yes

Reviewer #2: No

**- We will submit an Excel document as a supplemental document. This file contains the raw data used for this study.**

4. Is the manuscript presented in an intelligible fashion and written in standard English?

PLOS ONE does not copyedit accepted manuscripts, so the language in submitted articles must be clear, correct, and unambiguous. Any typographical or grammatical errors should be corrected at revision, so please note any specific errors here.

Reviewer #1: Yes

Reviewer #2: Yes

**5. Review Comments to the Author**

Please use the space provided to explain your answers to the questions above. You may also include additional comments for the author, including concerns about dual publication, research ethics, or publication ethics. (Please upload your review as an attachment if it exceeds 20,000 characters)

Reviewer #1: I do agree that evaluative studies at times, do not require IRB if there is no interaction or intervention with human subjects and or did not include any identifiable private information.
It is also, well noted that ordering for amylase as an adjunct to lipase in acute pancreatitis creates extra cost. However, increased amylase is seen in quite a number of conditions e.g., intestinal disease, pancreatic disease, decreased metabolic clearance and salivary disease. Thus, crawling toward obsolescence can perhaps be tagged to pancreatitis alone and not other notable conditions. At least, authors should insert a statement or two for this clarification.

I do agree with statements on lines 63 to 70. But again, reading through, it seems amylase obsolescence is just tagged to acute pancreatitis. Thus, prior statements are needed to consolidate this obsolescence in only acute pancreatitis.

- **We agree with the reviewer, and, in response, we have modified the title to indicate amylase utilization in the context of pancreatitis.**
  - **“Crawling toward obsolescence: The extended lifespan of amylase for pancreatitis.”**
- ***We agree with the importance for distinguishing amylase ordering for pancreatitis vs. other conditions. As such we have modified the discussion to indicate this.***
  - **Amylase utilization may never reach zero, as alternative indications for amylase exist, including salivary and ovarian tumors, ulcer, celiac disease, pregnancy, burns, and certain medications. Pancreatitis represent the most common reason for amylase testing, a practice identified as “a thing we do for no reason”**

Reviewer #2: The article is insightful and a good read. the following points are recommended:
*1. The authors are advised to check if the abstract format and referencing style is in line with the format of PLOS ONE.*

**-The abstract format has been adjusted**

2. Line 50 check grammar - 'changes in' instead of 'changes to'.	**-**

**Thank you for pointing this out. Change has been made**

3. Line 59 information on the serum amylase test could be added.

**- added a line at 61 for clarity*: Thus, at this point amylase became the standard test for diagnosis of pancreatitis.***
4. Line 72 more information on the serum lipase test could be added.

**- added a line at 74 -75 to signify the change of guard from amylase to lipase – “*Lott et al in 1991 suggested that lipase should replace amylase”***
5. Line 74-75 can be reframed for clarity.

**- We have rephrased this sentence for better clarity (Line 75-78)**

***Due to the longer half-life and higher peak concentrations of lipase as compared to amylase, lipase proved to be a superior test, especially in cases where the diagnosis was delayed.***

6. Line 76 the term 'subpopulation' may be replaced by 'subset' or other suitable term.

**- replaced subpopulation with “*subtype*” (line 79)**

7. Line 80 kindly check the relevance of [a] in the sentence.

**Response: We added the square brackets to signify a clarification to the quote. See the** [**APA style manual**](https://apastyle.apa.org/style-grammar-guidelines/citations/quotations/changes#:~:text=Use%20square%20brackets%2C%20not%20parentheses,follows%3A%20%5Bemphasis%20added%5D.)**.**

8. Line 107 The authors are requested to input the data for the utilization of creatinine and lipase over the period of study

**Response: From the first to the last year of the 14 year study period, creatinine testing changed by only 2.78 % (12,096,416 to 11,759,568) and lipase changed by 1.86% (303,570 to 297,920).**

*6. PLOS authors have the option to publish the peer review history of their article (*[*what does this mean?*](https://nam12.safelinks.protection.outlook.com/?url=https%3A%2F%2Fjournals.plos.org%2Fplosone%2Fs%2Feditorial-and-peer-review-process%23loc-peer-review-history&data=05%7C01%7Cnaga.kanaparthy%40yale.edu%7C520a6216d43f4ee85ba908dbe9e226d4%7Cdd8cbebb21394df8b4114e3e87abeb5c%7C0%7C0%7C638360929745966510%7CUnknown%7CTWFpbGZsb3d8eyJWIjoiMC4wLjAwMDAiLCJQIjoiV2luMzIiLCJBTiI6Ik1haWwiLCJXVCI6Mn0%3D%7C3000%7C%7C%7C&sdata=CWCHXb3Xdoj0%2BeuScug5P6DV%2BXiHgHjxSxfQ9VH4zfw%3D&reserved=0)*). If published, this will include your full peer review and any attached files.*

*While revising your submission, please upload your figure files to the Preflight Analysis and Conversion Engine (PACE) digital diagnostic tool,* [*https://pacev2.apexcovantage.com/*](https://nam12.safelinks.protection.outlook.com/?url=https%3A%2F%2Fpacev2.apexcovantage.com%2F&data=05%7C01%7Cnaga.kanaparthy%40yale.edu%7C520a6216d43f4ee85ba908dbe9e226d4%7Cdd8cbebb21394df8b4114e3e87abeb5c%7C0%7C0%7C638360929745966510%7CUnknown%7CTWFpbGZsb3d8eyJWIjoiMC4wLjAwMDAiLCJQIjoiV2luMzIiLCJBTiI6Ik1haWwiLCJXVCI6Mn0%3D%7C3000%7C%7C%7C&sdata=uX3YJWb3ZWUfXUT1pRGItYoK0VQqb2Zl9CBMxUlPiWc%3D&reserved=0)*. PACE helps ensure that figures meet PLOS requirements. To use PACE, you must first register as a user. Registration is free. Then, login and navigate to the UPLOAD tab, where you will find detailed instructions on how to use the tool. If you encounter any issues or have any questions when using PACE, please email PLOS at* [*figures@plos.org*](mailto:figures@plos.org)*. Please note that Supporting Information files do not need this step.*

**Fig 1 was uploaded and checked on the PACE too. The new file with a reduced size has been uploaded. Please note the initial file was .tiff and it was changed to .tif**
